# Probiotics, Prebiotics and Postbiotics on Mitigation of Depression Symptoms: Modulation of the Brain–Gut–Microbiome Axis

**DOI:** 10.3390/biom11071000

**Published:** 2021-07-07

**Authors:** Agata Chudzik, Anna Orzyłowska, Radosław Rola, Greg J. Stanisz

**Affiliations:** 1 Department of Neurosurgery and Paediatric Neurosurgery, Medical University of Lublin, Jaczewskiego 8, 20-090 Lublin, Poland; a.m.orzylowska@gmail.com (A.O.); radoslaw.rola@umlub.pl (R.R.); stanisz@sri.utoronto.ca (G.J.S.); 2 Independent Laboratory of Cancer Diagnostics and Immunology, Department of Oncological Gynaecology and Gynaecology, Medical University of Lublin, Staszica 16, 20-081 Lublin, Poland; 3 Physical Sciences, Sunnybrook Research Institute, 2075 Bayview Avenue, Toronto, ON M4N 3M5, Canada; 4 Department of Medical Biophysics, University of Toronto, 101 College Street, Toronto, ON M5G 1L7, Canada

**Keywords:** microbiota, brain–gut–microbiome axis, depression, probiotics, prebiotics, postbiotics

## Abstract

The brain–gut–microbiome axis is a bidirectional communication pathway between the gut microbiota and the central nervous system. The growing interest in the gut microbiota and mechanisms of its interaction with the brain has contributed to the considerable attention given to the potential use of probiotics, prebiotics and postbiotics in the prevention and treatment of depressive disorders. This review discusses the up-to-date findings in preclinical and clinical trials regarding the use of pro-, pre- and postbiotics in depressive disorders. Studies in rodent models of depression show that some of them inhibit inflammation, decrease corticosterone level and change the level of neurometabolites, which consequently lead to mitigation of the symptoms of depression. Moreover, certain clinical studies have indicated improvement in mood as well as changes in biochemical parameters in patients suffering from depressive disorders.

## 1. Introduction

The microbiome consists of the microorganisms (bacteria, archaea, viruses, protists and fungi), their genomes, and their surrounding environment, including the gastrointestinal tract, oral mucosa, urogenital and respiratory systems, and the skin surface [1]. Bacteria are the dominant group of microorganisms that make up the microbiome [2]. It is estimated that their number in the entire human body is of the same order as the number of human cells [3]. The majority of bacteria reside in the intestines [4]. Due to their huge number and variety, they can significantly affect normal physiology and modify the host’s susceptibility to diseases [5]. Multiple roles of bacteria in the gut include digestion, taking part in the production of short-chain fatty acids, vitamins synthesis, immune system maintenance, and influence on the permeability of the mucosal barrier [6,7]. Furthermore, additional administration of probiotic bacteria may provide health benefits to the host [5]. Amongst many different strains of probiotics, there are psychobiotic bacteria that have a beneficial effect on mental health when ingested in sufficient amounts [8]. It has been demonstrated that these bacteria have a significant impact on metabolism and central nervous system function, consequently influencing mental health [9,10], thanks to the brain–gut–microbiome (BGM) axis via neuronal, endocrine, and immune mechanisms [11,12]. The impact of probiotics on human neurometabolism can also be promoted by prebiotics, which stimulates the proper growth of probiotic bacteria and can support the gut–brain interaction [13]. Moreover, recent studies indicate the participation of postbiotics in the modulation of the BGM axis [14].

This review describes the properties of probiotics, prebiotics and postbiotics, focusing on their beneficial effect on human health in a regular diet. We discuss the ways through which the gut microbiota communicates with the brain. Furthermore, we focus on recent studies in both animals and humans investigating pro-, pre- and postbiotic supplementation in depressive disorders. We also discuss the shortcomings of these clinical trials. The increasing amount of research on the microbiome suggests that this is an extremely important issue and further investigations of the application of probiotics, prebiotics and postbiotics in the prevention and treatment of depression are essential.

## 2. Probiotics

The term “probiotic” originates etymologically from the words “pro bios” meaning “for life” and the beneficial effect of lactic acid fermentation products on human health has ancient roots [15]. The original theory is attributed to microbiologist and Nobel laureate Élie Metchnikoff, who was a co-author of many pioneering studies concerning the role and function of probiotic bacteria [16]. He assigns potential life-lengthening properties to lactic acid bacteria present in the human colon [16,17]. Nowadays, probiotics are defined as “live microorganisms that, when administered in adequate amounts, confer a health benefit on the host” [18]. These include mainly *Lactobacillus* and *Bifidobacterium* strains, as well as some *Streptococcus* and *Enterococcus* strains [18,19]. The beneficial effects associated with probiotics include antiallergic action [20], improvement of intestinal health (e.g., elimination of dysbiosis and sealing the intestinal epithelium) [21,22], enhancement of the immune response [23,24], inhibition of lactose intolerance [25], prevention of cancer [26,27], and a beneficial impact on mental health [28,29,30]. It should be emphasized that the majority of bacteria is first acquired at birth and is maintained and extended by diet [31,32]. In healthy people who maintain a healthy diet, there is a beneficial balance of microbiota. Otherwise, additional administration of probiotic bacteria may be considered [18]. Dinan et al. [8] have defined psychobiotics to be probiotics that, when ingested in adequate amounts, produce a health benefit in patients suffering from psychiatric illness [8]. In addition to a positive effect on intestines, psychobiotics contribute to changes in concentrations of brain neurotransmitters and proteins, reduction of cortisol levels, and alterations in serum cytokine levels, which consequently lead to behavioral changes, as demonstrated in animal and clinical studies [13]. 

Many bacteria can regulate neuroactive metabolites such as gamma-aminobutyric acid (GABA), 5-hydroxytryptamine (5-HT) and catecholamines that play an important role in brain and mental health [33,34,35]. GABA is an amino acid that inhibits synaptic conduction by hyperpolarization of neuronal cell membranes and consequently decreases activity in the central nervous system [33]. GABAergic system dysfunctions are strongly correlated with mood disorders [33]. It has been shown that depression and anxiety disorders are associated with decreased gamma-aminobutyric acid levels in the brain [36,37]. Among GABA-regulating bacteria, there are food-derived *Lactobacillus* strains like *Lactobacillus plantarum* [38,39,40,41], *Lactobacillus paracasei, Lactobacillus rhamnosus* [37,38,39,42], and *Lactobacillus brevis* [43,44,45,46,47,48]. Yunes et al. [49] have screened 135 human-derived *Bifidobacterium* and *Lactobacillus* strains for their ability to produce GABA. Some bacterial strains can also affect serotonin levels [50]. Serotonin is a monoamine neurotransmitter synthetized from tryptophan. Most 5-HT is produced in the enterochromaffin cells in the gastrointestinal tract [34]. The function of serotonin is very complex as it takes part in the regulation of mood, cognition, and several physiological processes [34]. A disrupted serotonergic system is one of the main causes of depression [34]. Bacteria strains that affect the 5-HT pathway are *Escherichia coli* [51], *Klebsiella pneumoniae*, *Morganella morganii* [52], *Lactobacillus plantarum*, *Lactococcus lactis subsp. cremoris*, *Streptococcus thermophilus* [53]. Bacteria that regulate catecholamines (adrenaline, noradrenaline and dopamine) include *Bacillus* spp., *Escherichia coli*, *Staphylococcus aureus* [50,51], *Klebsiella pneumoniae* and *Morganella morganii* [52]. Some of the these strains have been incorporated in improved health-promoting functional foods, which have a considerable effect on the regulation of neurometabolites [54].

## 3. Prebiotics

At first, prebiotics were defined as a “non-digestible food ingredient that beneficially affects the host by selectively stimulating the growth and/or activity of one or a limited number of bacteria already resident in the colon” [55]. Over time and with the advancement of our knowledge, however, the definition has been modified to include not only stimulation of bacteria residing in the colon but also other bacteria in the human body [56,57]. The International Scientific Association for Probiotics and Prebiotics proposes the following definition of prebiotic: “a substrate that is selectively utilized by host microorganisms conferring a health benefit” [58]. Prebiotics are nondigestible polysaccharides such as oligosaccharides, fructans (fructooligosaccharides, inulin) and galactooligosaccharides that can be found in many natural products and dietary ingredients [59] and are listed in Table 1. Bacterial fermentation of prebiotic carbohydrates results in the production of short-chain fatty acids (SCFAs) such as butyric acid, acetic acid or propionic acid [22]. Prebiotics and the SCFAs are crucial for intestinal health, stimulate the immune system, can be a source of energy for gut microbiota, and have antagonistic properties to detrimental gut bacteria [60,61,62]. Moreover, numerous experimental studies have proved that prebiotics can help decrease the severity of particular diseases such as mental disorders, diabetes, irritable bowel syndrome (IBS), infectious diseases and reduce the colon cancer risk [63,64]. 

## 4. Postbiotics

The term postbiotic has appeared in the literature for over 20 years, however, research on the effects of postbiotics has intensified over the last five years [81]. The International Scientific Association for Probiotics and Prebiotics proposed the following definition of postbiotics: “a preparation of inanimate microorganisms and/or their components that confers a health benefit on the host” [81]. The concept of non-living microorganisms that could promote or preserve health is not new, other terms that have been used to describe such substances include “paraprobiotics” [82,83], “heat-killed probiotics” [84,85], “metabiotics” [86] and “bacterial lysates” [87]. In order to provide a clear definition, a panel of experts have defined the scope of postbiotics to be deliberately inactivated microbial cells, with or without metabolites or cell components, that contribute to demonstrated health benefits [81]. They note that bacterial metabolites (e.g., lactic acid, proteins, vitamins, SCFAs) or cell components (including pili, cell wall components) would not qualify as postbiotics in their own right, although some might be present in postbiotic preparations [81]. Potential mechanisms for the mediation of health effects by postbiotics are similar to those of probiotics [14] and include enhancement of epithelial barrier function, modulation of host-microbiota, modulation of immune responses, modulation of systemic metabolism and signaling via the nervous system [81,88].

## 5. Brain-Gut-Microbiome Communication Routes

**Figure 1:**
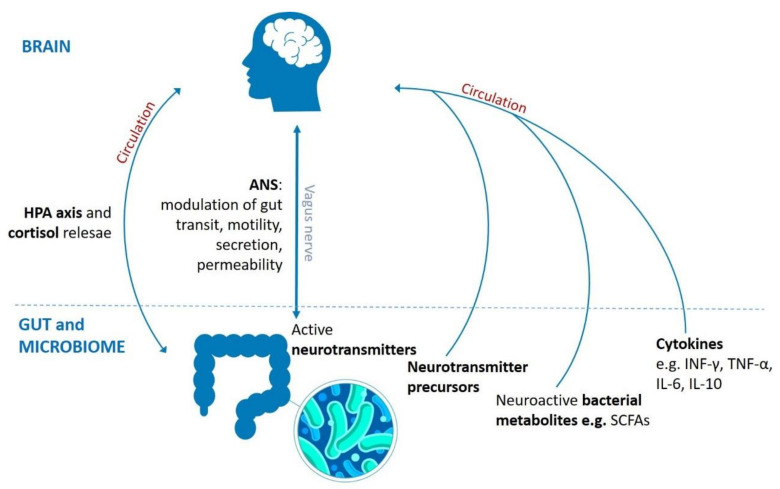
Routes involved in bidirectional communication pathway between brain, gut and gut microbiota. It comprises neuronal (vagus nerve, enteric nervous system, neurotransmitters), endocrine (HPA axis and stress hormones, e.g., cortisol), and immune (cytokines) mechanisms. HPA: hypothalamic–pituitary–adrenal axis, ANS: autonomic nervous system, SCFAs: short-chain fatty acids, CNS: central nervous system, INF-γ: interferon γ, TNF-α: tumor necrosis factor α, IL-6: interleukin 6, IL-10: interleukin 10.

Human organisms are under a significant stimulus from microorganisms inhabiting the intestines and from the metabolites produced by the microbiota [89,90]. Conversely, it is understood that the brain regulates the function of the gut and the structure of the gut microbial community via the autonomic nervous system by modulating intestinal transit, gut motility, secretion and gut permeability [91]. The bidirectional communication pathway between gut microbiota and the gut, and their interaction with the central nervous system, has been termed the brain–gut–microbiome (BGM) axis [11]. Investigations of the BGM axis involve animal studies in germ-free (GF) animals [92], prebiotic and probiotic studies in rodent models [93,94,95], probing the effects of antibiotics [96], fecal transplantation [97] and cultured gut organ systems [98]. These approaches allow for the identification of neuronal, neuroendocrine and neuroimmune routes of BGM mechanisms (Figure 1) [11]. 

### 5.1. Neuronal Routes 

The gut is innervated by the enteric nervous system (ENS), which is responsible for the coordination of intestinal function, e.g., motility, fluid secretion, blood flow and reaction to metabolites formed due to the activity of the intestinal microbiota [91]. The digestive function is controlled by the vagus nerve, pelvic nerve and sympathetic pathways [99]. Research to date suggests that specific bacterial strains may play a critical role in the development and function of the ENS, and exposure to bacteria at birth and during early life is essential for the postnatal development of the enteric nervous system [100]. For example, GF mice have been observed to have an immature intestinal nervous system and a deficit in sensory signaling, while reconstruction of their gut microbiota mitigates those shortcomings [101,102]. Moreover, both ex vivo [103] and germ-free animals studies [104] have shown that bacteria impact gut motility. Studies assessing changes in velocity, amplitude and frequency of contractions in the intact segments of jejunum and colon excised from mice have demonstrated that bacterial microvesicles, which are small lipids and bilayer structures secreted by bacteria, are mainly involved in the modulation of gut motility [105]. 

The vagus nerve plays a crucial role in communication between the gut and the brain [106]. Signals from the intestines are transmitted directly through the vagus nerve or indirectly through the mediation of enteroendocrine cells and hormonal factors [106]. Bravo et al. have established that dietary supplementation with lactic bacteria in healthy anxiolytic mice has a direct effect on GABA receptors in the CNS and reduces anxiety- and depressive-like behavior, while vagotomized mice do not show either neurochemical or behavioral effects [94]. This confirms the importance of the vagus nerve in the BGM pathway [94]. In addition, it has also been observed that vagotomized mice do not exhibit anxiety-like behavior associated with chronic colitis [93]. 

Numerous bacterial species present in the human intestine are capable of modulating neurotransmitter levels [50]. *Lactobacillus* and *Bifidobacterium* species have been found to produce GABA and histamine [49,107], while *Escherichia coli* produce serotonin, dopamine, and noradrenaline [51]. Neurometabolites produced by bacteria have the potential to influence the CNS and, consequently, behavior [50]. The mechanisms by which those molecules affect the brain involve vagus nerve signaling and circulation [11,108,109]. GABA, serotonin and dopamine cannot cross the blood–brain barrier (BBB) under normal physiological conditions [110,111,112]. However, some of the neurotransmitters’ precursors do cross the BBB and can then be transformed into active neurotransmitters [113,114]. This is the case with tryptophan, the precursor to serotonin, the availability of which is affected by gut bacteria [34]. It is also known that stress can activate enzymes of the kynurenine pathway, which can reduce the amount of tryptophan available for serotonin synthesis; in consequence, kynurenines are believed to play a significant role in the pathogenesis of depressive disorders [115,116]. 

The microbiome influences the concentrations of brain-derived neurotrophic factor (BDNF) in the brain [117]. This protein is a widely expressed neurotrophin serving several functions within the CNS, including neuronal differentiation and survival [118], and regulation of BDNF concentration is involved in depression and anxiety [119]. BDNF levels have been observed to be lower in the cortex and hippocampus of GF mice as compared to controls, suggesting that the gut microbiota contributes to the elevation of brain BDNF and may modulate behavior through changes in the BDNF level [92]. Moreover, recent research suggests that changes in the microbiome affect hippocampal neurogenesis and are dependent on age and gender [120,121,122].

### 5.2. Microbiota and the Hypothalamic–Pituitary–Adrenal Axis

One of the factors influencing the BGM axis along the endocrine pathway is the modulation of the hypothalamic–pituitary–adrenal axis (HPA) [123]. The HPA axis works on the principle of negative feedback and plays a key role in stimulating the body’s stress response and regulating physiological processes, including digestion, the functioning of the immune system, emotions and energy balance [123]. In response to stress in the humoral pathway, the hypothalamus releases corticotropin-releasing hormone (CRH). CRH reaches the pituitary gland via the circulation, which synthesizes adrenocorticotropic hormone (ACTH). ACTH stimulates the adrenal glands to synthesize glucocorticosteroid hormones (stress hormones), e.g., cortisol or corticosterone [124]. Acting systemically, stress hormones cause leakage of tight junctions and thus increase the permeability of the intestinal barrier [125]. This leads to bacterial translocation, which causes HPA axis response and immune activation [123,126]. The HPA axis response to acute stress can be alleviated by dietary probiotic supplementation [127]. It has been shown that the development of the HPA axis depends on postnatal microbial colonization [92]. Disturbances in the functioning of the HPA axis, increased susceptibility to stress and cognitive disorders were shown in germ-free mice [128].

### 5.3. Immune Routes

Immune mechanisms are relevant to the function of the BGM axis. The intestine and gut-associated lymphoid tissues (GALT) are the largest immune organs in the human body, providing a defensive barrier between external pathogens and the internal environment [129]. Immune cells, such as regulatory T cells (Tregs) and antigen-presenting cells (APC), control the gut and can be transferred from GALT to other peripheral lymphoid sites, including the CNS [130].

The intestinal microbiome modulates immune activity in two different ways [129]. Firstly, short-chain fatty acids and microbiota-derived bacterial fermentation products are produced by intestinal bacteria. These can then be transported to the brain and have a direct effect on neuronal cells and immune cells such as microglia [131]. However, the mechanism by which these molecules cross the blood–brain barrier is not fully understood [129]. It is known that dietary bacterial metabolites such SCFAs may pass from the intestines into the systemic circulation, where they interfere with immune regulation and CNS function [132]. Moreover, it has been reported that butyrate (a short-chain fatty acid) modulates brain function by inhibiting histone deacetylase, and its administration was found to have an antidepressant effect by inducing histone hyperacetylation in mice [133]. 

Secondly, the gut microbiome influences peripheral immune cells, which then transmit signals to the brain via cytokines [134], which play a crucial role in neurodevelopment and neuroinflammation [135,136,137]. Additionally, several studies confirm that the microbiome modulates the concentration of anti-inflammatory cytokines and lowers the levels of pro-inflammatory cytokines, e.g., interferon γ (IFN-γ) and tumor necrosis factor α (TNF-α) [138]. This effect has been demonstrated in rats supplemented with *Bifidobacteria*, which contributes to reduced levels of IL-6, INF-γ and TNF-α, as compared to the placebo group [12].

Considering the significance of the brain–gut–microbiome axis in the functioning of the organism, there is an increasing number of studies describing the beneficial effects of probiotics and prebiotics on mental health, the majority of which are preclinical studies in animal models of disease. Below we present recent reports on the use of probiotics and prebiotics in studying depression disorders.

## 6. Depression

According to the WHO, over 300 million people worldwide suffer from depression [139]. The main symptoms of depression include affective disorders such as sadness, anhedonia, the impression of reduced intellectual performance, cognitive disorders and low self-esteem [140]. Depressed patients display apathy and reduced motivation, withdrawing from social activity and limiting their typical behavior. Somatic symptoms are dominated by changes related to disturbance of sleep, appetite, and energy levels [141]. To explain the pathophysiological mechanisms of depression, various hypotheses including monoamine, genetic, environmental, immunologic, endocrine factors and neurogenesis have been proposed, but the full elucidation of the depression pathophysiology remains challenging [141,142]. Taking into account the existence of the BGM axis, there is growing scientific evidence for a strong link between depressive disorders and the gut microbiome [10,143]. Many studies show the positive effect of probiotics and prebiotics as adjuvant treatment of depressive disorders [144,145]. Conversely, several studies have shown reduced microbiota diversity in patients suffering from depression [143]. Most up-to-date findings from the preclinical and clinical trials of the use of probiotics and prebiotics in depressive disorders are described below and listed in Table 2 and Table 3. The herein mentioned animal studies focus mainly on the adolescent age span. Adolescence comprises the most important period of postnatal neurodevelopment [146]. Considering the multitude of ongoing neurodevelopmental processes in the adolescent brain, it should be noted that most adult neuropsychiatric disorders have their roots exactly during this time span [146,147].

### 6.1. Probiotic Studies

#### 6.1.1. Animal Studies

Studies show that dietary supplementation with probiotic bacteria increases the level of neurotransmitters in brain tissues and has a potentially beneficial effect on the prevention and treatment of depression [148,150]. The antidepressant effect of *Lactobacillus plantarum* DP189 (DP189) isolated from Chinese traditional fermented sauerkraut has been demonstrated in Sprague Dawley rats subjected to a corticosterone-induced model of chronic stress [148]. Rats were orally gavaged for 21 days; followed by behavioral, histopathological and biochemical studies to assess changes in comparison with a control group, and with rats treated with fluoxetine (serotonin re-uptake inhibitor) [148]. Behavioral Morris water maze and sucrose preference tests have shown that the DP189 supplementation improves memory and spatial learning and decreases anhedonia [148]. Similarly, Barros-Santos et al. have used other *L. plantarum* strains isolated from fermented food and observed behavioral changes manifested in antidepressant- and anxiolytic-like effects in healthy male mice [149]. *L. plantarum* DP189-treated rats show decreased serum IL-1β and TNF-α concentrations and, histopathologically, lower levels of hippocampal apoptosis of neurons than the stress group [148]. Moreover, the stress group exhibited decreased hippocampal levels of serotonin (5-HT), dopamine (DA), and norepinephrine (NE), which were alleviated by the DP189 supplementation [148]. A similar effect of probiotic treatment has been observed in studies on the same model of depression in mice [150]. The researchers have tested the antidepressant-like effect of live and heat-killed (postbiotic) *Lactobacillus paracasei* PS23 (PS23) [150]. In open-field and sucrose preference tests, they show that both live and heat-killed PS23 are able to reverse chronic corticosterone-induced anxiety- and depressive-like behaviors and reverse corticosterone-reduced BDNF protein levels in the hippocampus [150]. Furthermore, mice treated with live bacteria exhibited elevated serotonin levels in the hippocampus, prefrontal cortex and striatum, compared to the stress group [150]. In turn, the heat-killed bacteria increased dopamine levels in the hippocampus and prefrontal cortex [150]. The authors emphasized that both live and heat-killed PS23 can reverse chronic corticosterone-induced anxiety- and depression-like behaviors, moreover, they note that live probiotics could influence the gastrointestinal microbiota and have immunomodulatory effects, while the components of dead probiotics may exert an anti-inflammatory response [150]. In a study by Janik et al., adult male BALB/c mice were treated with *Lactobacillus rhamnosus* JB-1-enriched diet (1 × 10^9^ CFU daily for four weeks), and in vivo magnetic resonance spectroscopy (MRS) was used to measure cerebral levels of neurometabolites [168]. Glutamate and glutamine, GABA and N-acetylaspartate (NAA) levels increased after four weeks of bacterial diet compared to baseline concentrations [168]. Changes in glutamate and GABA were confirmed using an enzyme-linked immunosorbent assay (ELISA) [168]. A related study that also involved MRS measurements has shown that dietary supplementation with the same *Lactobacillus rhamnosus* strain reverses stressed-induced decreases in brain metabolites [37]. In this study, a Wistar rat model of chronic unpredictable mild stress (CUMS) was used. After the stress procedure, the rats were fed a microbiotic diet for four weeks (1.7 × 10^9^ CFU daily by oral gavage) [37]. The elevated plus maze behavioral test showed that treatment with *L. rhamnosus* JB-1 bacteria resulted in the reduction of stress-induced behavior and restored the levels of GABA, glutamate + glutamine, total N-acetylaspartate and total creatine to concentrations observed in the control group [37]. Another probiotic bacterial strain, *Bifidobacterium breve* CCFM1025 (CCFM1025), has been investigated in a CUMS model in C57BL/6 mice [151]. After five weeks of oral CCFM1025 supplementation, the mice demonstrated decreased anxiety- and depressive-like behaviors [151]. In addition, the bacterial treatment mitigated hypothalamic–pituitary–adrenal (HPA) axis hyperactivity-induced inflammation: serum corticosterone concentrations were elevated in stressed mice, while all these abnormalities were restored by the treatment with CCFM1025 [151]. Strengthening of the serotonergic system in the gut and brain increased expression of BDNF in the hippocampus—modification of the gut microbial composition and metagenome have also been observed [151]. Elevated brain-derived neurotrophic factor expression in the hippocampus along with reduced expression of TNF-α, interleukin-1b (IL-1b), nuclear factor kappa B (NF-kB) and Iba1 protein, as well as anxiolytic and antidepressant effects in behavioral tests were observed after *Bifidobacterium adolescentis* treatment in a chronic restraint stress (CRS) model in mice [152]. 

#### 6.1.2. Human Studies

Wallace and Milev conducted an open-label pilot study on 10 patients with a current episode of major depressive disorder (MDD) who were not taking any antidepressant drugs [158]. Patients received probiotic supplementation with *Lactobacillus helveticus* R0052 and *Bifidobacterium longum* R0175 (CEREBIOME^®^) at a dose of 3 × 10^9^ CFU once a day for 8 weeks [158]. Clinical data were measured at baseline, week 4, and week 8 using a validated series of clinical scales and self-report questionnaires from the Canadian Biomarker Integration Network in Depression (CAN-BIND) [158]. The results showed that daily supplementation with probiotics significantly reduced anxiety and improved overall mood and anhedonia by week 4 and sleep quality by week 8 [158]. In contrast, a double-blind pilot randomized controlled trial [159] including 40 pregnant women with uncomplicated pregnancies and elevated depressive symptoms and/or anxiety, who orally consumed a probiotic multispecies mixture once a day (Ecologic Barrier; 2.5 × 10^9^ CFU/g) from 26 to 30 gestation weeks until delivery showed no differences in mood between probiotic-supplemented and placebo groups, which may have been related to the demanding inclusion and exclusion criteria of the participants [159]. In another double-blind, randomized, placebo-controlled study, Rudzki et al. investigated the properties of *Lactobacillus plantarum 299v* (*LP299v*) [160]. In addition to a validated series of clinical scales and self-report questionnaires, biochemical parameters were assessed [160]. Seventy-nine patients with MDD took part in this study, and sixty completed it [160]. The participants received either a selective serotonin reuptake inhibitor (SSRI) with probiotic *LP299v* (10 × 10^9^ CFU daily) (n = 30) or SSRI with a placebo (n = 30) for 8 weeks [160]. There were no significant changes in the IL-6, IL-1b, TNF-α, and cortisol levels in either the probiotic or placebo groups. However, a significant decrease in the kynurenine concentration and improvement of cognitive functions was shown in the *LP299v* group as compared to the placebo group with subsequent improvement of cognitive functions [160]. This study indicates the advisability of enriching standard depression therapy with specific strains of probiotic bacteria [160]. The effect of probiotic and prebiotic supplementation on circulating proinflammatory cytokines and urinary cortisol levels has also been studied by Kazemi et al. [164]. They conducted a double-blind placebo-controlled randomized clinical trial in 110 participants diagnosed with MDD who had been taking antidepressant medications for at least 3 months prior to the trial [164]. They were randomly divided into three groups and received ⩾10 × 10^9^ CFU of *L. helveticus* R0052 and *B. longum* R0175 or galactooligosaccharide and 0.2% plum flavor as a prebiotic or placebo for 8 weeks (along with antidepressant drug treatment) [164]. The serum inflammatory cytokines levels were not altered in any groups [164]. However, the probiotic supplementation reduced depression symptoms as measured with the Beck Depression Inventory (BDI) score, and decreased the urinary cortisol levels relative to the control group were observed [164]. Another probiotic strain, *Bacillus coagulans* MTCC 5856, was tested for its efficiency in MDD in irritable bowel syndrome (IBS) patients [161]. Forty patients, randomly divided into two equal groups, received either the probiotic at a daily dose of 2 × 10^9^ CFU or a placebo for 90 days [161]. Effects on the clinical symptoms of MDD measured pre- and post-intervention with the list of clinical scales and self-report questionnaires, and biochemical parameters were also assessed [161]. The study indicates that *Bacillus coagulans* MTCC 5856 supplementation significantly reduces clinical depression symptoms as measured by scores of primary efficacy tests (e.g., Hamilton depression rating scale, HDRS) [161]. In addition, the probiotic strain demonstrated a beneficial effect on sleeplessness and decreased the level of myeloperoxidases, which are suggested to regulate the production of free radicals leading to cellular oxidative stress and, consequently, linked with depression and some neurodegenerative diseases [161]. 

### 6.2. Prebiotic Studies

#### 6.2.1. Animal Studies

The literature showing the effects of prebiotics on depressive disorders is much less abundant than the research on probiotics. Chi et al. [153] investigated the antidepressant efficacy of fructo-oligosaccharides (FOSs) extracted from *Morinda officinalis* How., a traditional Chinese herb. They used a chronic unpredictable mild stress model of Sprague Dawley rats and then have introduced FOSs via intragastric gavage [153]. The antidepressive properties were assessed through behavioral tests, intestinal morphology and measurements of corticosterone levels [153]. Additionally, the bacterial genomic DNA from feces was extracted and gut microbiota profiling was carried out [153]. In an open-field test and sucrose preference test, it was reported that the FOS treatment alleviated depression-like behaviors [153]. Rats with CUMS showed activation of the HPA axis, as evidenced by elevated levels of corticosterone. The FOS treatment restored plasma and urine levels of corticosterone to levels observed in control rats and may have repaired the damage to the intestinal epithelium [153]. The gut microbiota profile in the CUMS rats indicated the process of dysbiosis. Compared to the control rats, the percentage of bacteria in the gut belonging to *Barnesiella, Acinetobacter, Coprococcus, Lactobacillus* and *Dialister* was reduced, while depression-associated bacteria were present (e.g., *Anaerostipes, Streptococcus, Proteobacteria*) [153]. It was shown that FOS supplementation promoted the presence of beneficial bacterial strains associated with antidepressant properties [153]. In yet another study, a specific mix of non-digestible galactooligosaccharides (BGOS) was investigated [154]. Male CD1 mice were fed BGOS for 3 weeks, and then anxiety was induced through a single injection of lipopolysaccharide (LPS) [154]. Subsequently, behavior, cytokine expression, serotonin and serotonin byproduct, and 5-hydroxyindole acetic acid (5-HIAA) levels were assessed and compared to the control group [154]. In the light–dark box behavioral test, BGOS-fed mice were observed to be less anxious than those from the control group, but the effect on locomotor activity was ambiguous [154]. A similar relationship was observed for serotonin receptors such 5-HT2A, but not for the 5-HT1A serotonin receptor, NMDA receptor subunits, or 5-HIAA [154]. As mentioned above, both prebiotics, i.e., fructo-oligosaccharides and galacto-oligosaccharides, have potentially antidepressant or anxiolytic properties [153,154]. Burokas et al. [155] investigated supplementation with FOS and GOS as well as a combination of FOS+GOS in C57BL/6J male mice. The probiotics were administered to healthy mice for 3 weeks prior to measurements of corticosterone and SCFA levels, assessment of behavior, and determination of gut microbiota composition [155]. Additionally, the effects of the FOS+GOS treatment in a CUMS model were tested [155]. A series of behavioral tests evaluating anxiolytic and antidepressant properties of the treatment were carried out (open-field, elevated plus maze, defensive marble burying, stress-induced hyperthermia, three-chamber test, female urine sniffing test, novel object recognition test, tail suspension test, hot plate, forced swim test) [155]. The study confirmed that these prebiotics significantly altered the behavior and neurochemistry associated with anxiety and depression in mice. Supplementation with FOS+GOS yielded both antidepressant and anxiolytic outcomes [155]. Moreover, it correlated with lower stress-induced plasma corticosterone and L-tryptophan concentrations, which was especially evident in the FOS+GOS combination and with monoamine level alterations [155]. The hippocampal mRNA levels of BDNF and the GABA(B) subunit receptor were also increased in animals administered with the FOS+GOS combination [155]. Changes in gene expression in the hippocampus were also observed by Neufeld et al. after prebiotic supplementation, as compared to control animals [169]. Furthermore, levels of short-chain fatty acids following FOS+GOS supplementation strongly correlated with positive behavioral effects [155]. Finally, microbiota composition exhibited a decreased *Actinobacteria/Proteobacteria* ratio after stress, which was normalized by prebiotic supplementation [155].

#### 6.2.2. Human Studies

Similar changes in the composition of the gut microbiome has been observed in patients with depression [170]. Several studies also show positive neurobehavioral effects of the combination of probiotics and prebiotics in the treatment of depression disorders [169,171]. In a clinical trial, Ghorbani et al. [163] investigated the effects of specific probiotics and a fructo-oligosaccharide prebiotic (Familact H^®^) as an adjuvant therapy to fluoxetine. Patients received fluoxetine (20 mg/d) for four weeks before entering the study. In this double-blind, multicenter trial, 40 patients with moderate depression were assessed [163]. The main measure outcome score of the HDRS (Hamilton Depression Rating Scale) was reported [163]. Then patients were randomly divided into a Familact group, which was given two capsules of the symbiotic (plus fluoxetine) for six weeks and a placebo group receiving placebo capsules (plus fluoxetine) for the same time [163]. The study showed that the pro- and prebiotic group had significantly decreased HDRS scores compared to the placebo group, which emphasizes the usefulness of the symbiotic as a complementary therapy [163]. In contrast, another randomized clinical trial did not reveal any significant effects of prebiotic supplementation in patients with MDD who took antidepressant drugs (sertraline, fluoxetine, citalopram, or amitriptyline) for at least three months before the trial [165]. In this study, 110 patients were divided into three groups-receiving a probiotic (*Lactobacillus helveticus* and *Bifidobacterium longum*), a prebiotic (galactooligosaccharides) or a placebo for eight weeks (all patients received antidepressant drugs simultaneously) [165]. While the supplementation of patients with MDD with the probiotic improved the Beck Depression Inventory (BDI) score in comparison with the group receiving the placebo, the prebiotic supplementation did not influence the BDI scale results in severe cases. Results may have been affected by the fact that the antidepressant drugs the participants had taken were not identical. [165]. Vaghef-Mehrabany et al. [162] have conducted a study of prebiotics combined with calorie restriction on metabolic and clinical response in 62 obese women with major depressive disorder. Half of the patients received an inulin prebiotic (10 g/day) and the other half were treated with placebo (maltodextrin, 10 g/day) for eight weeks [162]. Additionally, all the participants were on a 25% calorie-restricted diet [162]. Depression symptoms were evaluated by HDRS and Beck Depression Inventory (BDI-II) scales before and after the intervention, and anthropometric and biochemical parameters were also evaluated [162]. There were no statistically significant differences in depression symptoms between the prebiotic and placebo groups after the supplementation [162]. However, the study shows that while the administration of the prebiotic had little effect on metabolic changes, the reduced calorie content and subsequent weight loss had a more significant effect on the improvement of the well-being of obese patients with MDD [162].

### 6.3. Postbiotic Studies

#### 6.3.1. Animal Studies

Studies showing the beneficial effects of postbiotics on mental health are limited as little attention has been paid to the potential influence of non-viable bacterial cells and their components on the gut–brain interaction. However, some recent studies demonstrate the beneficial effects of postbiotics on depression. The antidepressant effects of heat-killed *Lactobacillus helveticus* strain MCC1848 in a mouse model of subchronic and mild social defeat stress (sCSDS) were investigated [156]. Heat-killed cells were prepared by treatment at 90 °C for 15 min. The dose of bacteria was 1.0 × 10^9^ organisms/day for 24 days [156]. Series of behavioral tests, fecal microbiota analysis and gene expression profiles in the nucleus accumbens were performed [156]. Significantly increased interaction time in the social interaction test and sucrose preference ratio in the sucrose preference test were observed, indicating anxiolytic- or antidepressant-like effects in the sCSDS mouse model. Additionally, gene expression in the nucleus accumbens, which is a stress-relevant brain region, was modulated by this postbiotic [156]. Anti-anxiety effects of heat-killed bacteria were also observed in healthy rodents [85,157]. Male C57BL/6J mice were fed with heat-killed *Enterococcus faecalis* strain EC-12 (EC-12) (diet enriched with 0.125% concentration of heat-killed EC-12 for 4 weeks) [85]. Behavioral tests (open-field, elevated plus-maze and forced swim tests) showed that mice supplemented with EC-12 had decreased anxiety-like and depression-like behavior [85]. Moreover, EC-12 supplementation modified the gene expression profile in the prefrontal cortex and significantly increased *Butyricicoccus* and *Enterococcus* composition in the gut compared to the control group [85]. Warda et al. investigated the postbiotic ADR-159, containing a co-fermentate of heat-killed *Lactobacillus fermentum* and *Lactobacillus delbrueckii* on male C57BL/6 mice [157]. ADR-159 was incorporated into standard mice chow to a concentration of 5% (3 × 10^9^ cell bodies/gram of chow) for three weeks [157] followed by a battery of behavior tests, and measurement of microbiota concentrations and corticosterone levels. They show that postbiotic ADR-159 diet supplementation subtly but distinctly changed the composition of the microbiota, decreasing locomotor activity in the open-field test and reducing the baseline corticosterone levels, which may indicate action on the HPA axis [157]. Although both studies are promising, further studies in depression models are required to elucidate the exact mechanisms of the effect of the heat-killed bacteria on the brain.

#### 6.3.2. Human Studies

In humans, the efficacy and health benefits of the long-term supplementation with heat-inactivated, washed *Lactobacillus gasseri* CP2305 (CP2305) was investigated in a group of 60 young adult students preparing for the national examination for medical practitioners (this model has been used in multiple studies of chronic psychological stress and is considered appropriate) [166]. In a double-blind, placebo-controlled, parallel-group clinical trial, 41 men and 19 women ingested CP2305-containing (1 × 10^10^ bacterial cells pre 2 tablets) or placebo tablets once daily for 24 weeks [166]. With the use of questionnaires assessing mental and physical states, the trial demonstrated that CP2305 significantly reduced anxiety and sleep disturbance compared to placebo. Moreover, fecal microbiota analyses show that diet supplementation with CP2305 attenuated the stress-induced decline of *Bifidobacterium* spp. and the stress-induced elevation of *Streptococcus* spp. [166]. While researchers noted the beneficial impact of *Lactobacillus gasseri* CP2305 for young adults experiencing stressful conditions in this study, the mechanism underlying the stress-relieving effects remains unclear and future studies are needed [166]. In another study, the effects of the postbiotic *Lactobacillus paracasei* MCC1849 (LAC-Shield™) supplementation on mood and symptoms of the common cold in healthy adults was shown [167]. Two hundred forty-one participants were randomized to receive 1 × 10^10^ heat-killed *L. paracasei* MCC1849 cell powder (10LP), 3 × 10^10^ heat-killed *L. paracasei* MCC1849 cell powder (30LP), or a placebo once a day for 12 weeks [167]. They demonstrated in the profile of mood states 2 (POMS 2) questionnaire (standard validated psychological test used to assess transient mood states) that the level of deterioration in the positive mood state caused by stress was decreased in the MCC1849-intake group relative to the placebo group. Moreover, no adverse effects associated with the postbiotic supplementation were observed during the study [167].

## 7. Conclusions and Future Directions

The data presented provide ample evidence for the beneficial role of probiotics, prebiotics and postbiotics on the brain-gut-microbiota axis in animal models of depression. In clinical studies, probiotics demonstrated a greater potential than prebiotics or postbiotics in reducing symptoms of depression in patients. More studies are needed to fully evaluate the therapeutic potential of pre- and postbiotics. Though most of the studies mentioned focused mainly on live probiotics, the use of inanimate microorganisms may have some attractive advantages, including a long shelf-life for commercial products and relatively easy standardization. Nevertheless, because of the novelty of postbiotic interventions, their safety and potential dangers remain poorly understood, hence, further research is necessary. 

Although animal studies in the field of pro-, pre- and postbiotics are promising, clinical trial results are slightly disappointing. For this reason, certain considerations should be taken. Initially, the small sample size of some clinical studies undermines the statistical interpretation and generalizability [158,159,161,163]. Demanding inclusion and exclusion criteria [159,161] or various degrees of depression, and the type of basic treatment [164,165] may also be responsible. It is worth noting that changes are compared in groups and not in individual study participants, which may also affect the results. Additionally, while each study includes a validated series of clinical scales and self-report questionnaires, not all of these clinical trials included biochemical measurements, which could improve interpretation of the results [158,159,163]. Another issue is the use of a single bacterial strain [160,161]. Due to the specific mechanisms of bacterial activity, it could be beneficial to test a multi-species probiotic or a synbiotic, which could result in a better therapeutic effect. Furthermore, the interactions of the administered bacterial strains and prebiotics with the native microbiota and its variability may also affect the reactions of patients to the treatment. In the future, it would be reasonable to consider fecal microbiome analysis, which has not been included by any of the studies discussed here. Also missing is the duration of the clinical effects and whether the administration of the treatment could be extended. Therefore, clinical trials with extended follow-up durations are needed. Finally, this research supports the importance of possible new therapeutic targets in the field of nutritional neuropsychopharmacology, however, more clinical trials are required.

## Figures and Tables

**Table 1:** Prebiotic sources.

Prebiotic	Source	Reference
Fructooligosaccharides (FOS)	Asparagus, Jerusalem artichoke, chicory, the blue agave plant, wheat, garlic, onion	[65,66,67]
Inulin	Chicory, Jerusalem artichoke, garlic, asparagus, onion, yacon	[65,68]
Galactooligosaccharides (GOS)	Milk, lentil, *Lycopus lucidus* herb	[69,70,71]
Xylooligosaccharide (XOS)	Bamboo shoots, honey, milk, rice, corn cob	[72,73]
Mannooligosaccharides (MOS)	Palm kernel products	[74,75]
Resistant starch	Cereal grains, seeds, legumes, starchy fruits and vegetables	[76,77,78]
Soybean-oligosaccharide (SOS)	Soybean	[79]
Lactulose	Milk	[80]

**Table 2:** Probiotics, prebiotics and postbiotics in animal studies on models of depression disorders.

Model	Probiotic/Prebiotic/ Postbiotic	Dosage, Route of Administration, and Length of Treatment	Test	Main Behavioral and Physiological Outcomes	Reference
**Probiotic studies**					
Corticosterone-induced chronic stress, adolescent male Sprague Dawley rats, N = 10/group	*Lactobacillus plantarum* DP189	1.0 × 10^9^ CFU/0.2 mL/day, oral gavage, 21 days	SPT, FST, Morris test, brain monoamines and proteins, serum cytokines, histopathology, hippocampus apoptosis	memory and spatial learning ↑ anhedonia ↓ IL-1β and TNF-α ↓ 5-hydroxytryptamine ↑ dopamine ↑ hippocampal mitogen-activated protein kinase 7 and c-Jun N-terminal kinase 2 ↓ down-regulation of pro-apoptosis protein Bax immunocontent up-regulation of antiapoptotic protein Bcl-2 immunocontent apoptosis of hippocampal cells ↓ hippocampal pathological changes ↓	[148]
Adult male Swiss mice, N = 16/group	*Lactobacillusplantarum* 286 and *Lactobacillus plantarum* 81	*L. plantarum* 286: 10^9^ CFU/0.1 mL /day *L. plantarum* 81: 10^9^ CFU/0.1 mL /day oral gavage, 30 days	OFT, FST, PM-DAT	*L. plantarum* 286 but not *L*. *plantarum* 81: anxiety- and depression-like behavior ↓	[149]
Corticosterone-induced depression model, adolescent male C57BL/6J mice, N = 8/group	*Lactobacillus paracasei* PS23 live or heat-killed	10^8^ CFU/0.2 mL/day oral gavage, 40 days	OFT, FST, SPT, Brain monoamines and proteins, serum corticosterone	Live: serotonin in hippocampus, prefrontal cortex and striatum ↑ Heat-killed: BDNF ↑ Both: mineralocorticoid, and glucocorticoid receptors ↑ anxiety- and depression-like behavior ↓	[150]
Chronic unpredictable mild stress, adolescent male Wistar rats, N = 19/group	*Lactobacillus rhamnosus* JB-1 (LR-JB1™)	1.7 × 10^9^ CFU/ 0.2 mL/day oral gavage, 4 weeks	EPM, brain metabolites level in MR spectroscopy	stress-induced behavior ↓ glutamate ↑ GABA ↑ glutamate + glutamine ↑ total N-acetylaspartate ↑ total creatine ↑	[37]
Chronic unpredictable mild stress, adolescent male C57BL/6J mice, N = 10/group	*Bifidobacterium breve* CCFM1025	10^9^ CFU/mL 0.1 mL/10 g body weight, oral gavage, 5 weeks	FST, TST, EPM, OFT, SPT, brain monoamines and proteins, serum corticotropin -releasing factor (CRF), corticosterone, inflammatory cytokines, SCFAs, fecal microbial composition	depression- and anxiety-like behaviors ↓ expression of BDNF in hippocampus ↑ SCFAs ↑ 5-HTP ↑ corticosterone ↓ modification of gut microbial composition and metagenome	[151]
Chronic restraint stress, adolescent male ICR mice, N = 12/group	*Bifidobacterium adolescentis*	0.25 × 10^9^ CFU/kg gavage, 21 days	OFT, EPM, TST, FST, brain protein and inflammatory cytokines, cecal microbial composition	depression- and anxiety-like behaviors ↓ BDNF expression in hippocampus ↑ inflammatory cytokines expression in hippocampus ↓ reverse the imbalance of cecal microbiota induced by CRS	[152]
**Prebiotic studies**					
Chronic unpredictable mild stress, adolescent male Sprague Dawley rats, N = 12/group	Inulin-type fructo-oligosaccharides (FOSs) extracted from *Morinda officinalis*	50 mg/kg oral gavage, 3 weeks	SPT, OPT, urine and plasma corticosterone, histopathology, fecal microbial composition	anhedonia-like behavior ↓ locomotor activity levels ↓ corticosterone in plasma and urine ↓ reparation of damages of intestinal epithelium changes in fecal microbial composition	[153]
Lipopolysaccharide (LPS)-induced anxiety, Adolescent male CD1 mice, N = 15/group	Specific mix of nondigestible galacto-oligosaccharides (Bimuno^®^, BGOS)	13 g of BGOS powder/1L of water, administration via drinking water, 3 weeks	LMA, MBT, light–dark box, brain monoamines and cytokines, 5-HT receptors and NMDAR subunits	anxious behavior ↓ IL-1b and 5HT2A receptors stabilization	[154]
Chronic psychosocial stress, adolescent male C57BL/6J mice N = 10/group	Fructo-oligosaccharides (FOS) and galacto-oligosaccharides (GOS),	dissolved in drinking water for 0.3–0.4 g/mouse/day, 3 weeks	3-CT, FUST, OFT, NOR, MBT, EPM, SIH, TST, RIT, FC, HP, FST, plasma corticosterone and tryptophan, brainneurotransmitters and proteins, spleen cytokine, cecum SCFAs, cecalmicrobiota composition	depression- and anxiety-like behaviors ↓ stress-induced corticosterone relesae ↓ cecal acetate and propionate ↑ cecal isobutyrate ↓ corticosterone ↓ proinflammatory cytokine ↓ L-tryptophan ↓ BDNF expression in the hippocampus ↑ changes in microbiota composition	[155]
**Postbiotic studies**					
Subchronic and mild social defeat stress (sCSDS), adolescent male C57BL/6J (B6) mice N = 16	Heat-killed *Lactobacillus helveticus* strain MCC1848	1.0 × 10^9^ organisms/day, 24 days	SIT, NBT, SPT, TST, FST, microbiota composition, gene expression profiles in the nucleus accumbens	depression- and anxiety-like behaviors ↓ modulation of gene expression in the nucleus accumbens	[156]
Adult male C57BL/6 J N = 8	Heat-killed *Enterococcus fecalis* (EC-12)	Diet enriched with 0.125% concentration of heat-killed EC-12, 4 weeks	OFT, EPM, FST, gene expression profile in the prefrontal cortex, plasma corticosterone, microbiota composition	depression- and anxiety-like behaviors ↓ modulation of gene expression profile in the prefrontal cortex *Butyricicoccus* and *Enterococcus* composition in the gut ↑	[85]
Adult male C57BL/6 mice	ADR-159 contains a heat-killed *Lactobacillus fermentum* and *Lactobacillus delbrueckii*	3 × 10^9^ cell bodies per gram of chow, 3 weeks	OF/NOR, MB, EPM, C, TST, FST, microbiota composition, plasma corticosterone	calming’ or sedative behavior effect corticosterone ↓ subtly changes in the composition of the microbiota	[157]

↑: increase of the measured parameter; ↓: decrease of the measured parameter. SPT: sucrose preference test; OFT: open-field test; FST: forced swim test; PM-DAT: plus maze-discriminative avoidance test, TST: tail suspension test, EPM: elevated plus maze, 3-CT: three-chamber test, FUST: female urine sniffing test, NOR: novel object recognition test, RIT: resident-intruder test, HP: hot plate, FC: fear conditioning; SCFAs: short-chain fatty acids; SIT: social interaction test; NBT: nest building test; OF/NOR: open-field/novel object recognition; MB: marble burying; C: Carmine red.

**Table 3:** Probiotic, prebiotics and postbiotics in human studies.

Study Design	Probiotic/Prebiotic/ Postbiotic	Dosage, Route of Administration, and Length of Treatment	Measures	Outcomes	Reference
**Probiotic studies**					
10 patients with a current episode of MDD, open-label exploratory study	*Lactobacillus helveticus* R0052 and *Bifidobacterium longum* R0175 (CEREBIOME^®^)	3 × 10^9^ CFU/ day, 8 weeks	CAN-BIND, MADRS, QIDS-SR16, SHAPS, GAD-7, STAI, PSQI	depressive symptoms↓ anxiety ↓ overall mood ↑ sleep quality ↑	[158]
40 pregnant women with low-risk pregnancies and elevated depressive symptoms and/or anxiety, probiotic group (N = 20), placebo group (N = 20), double-blind pilot randomized controlled trial	Probiotic multispecies mixture: Ecologic Barrier (*Bifidobacterium* *bifidum* W23, *Bifidobacterium lactis* W51, *Bifidobacterium lactis* W52, *Lactobacillus acidophilus* W37, *Lactobacillus brevis* W63, *Lactobacillus casei* W56, *Lactobacillus salivarius* W24, *Lactococcus* *lactis* W19 and *Lactococcus lactis* W58)	2.5 × 10^9^ CFU/g × 2/day, from 26 to 30 weeks gestation until delivery	EPDS, LEIDS-R, PRAQ-R, STAI, PES, MAAS, MAPS, PSQI	no significant differences between the probiotic and placebo groups in depression symptoms	[159]
60 patients with MDD, probiotic group (N = 30), placebo group (N = 30), double-blind, randomized, placebo-controlled study	*Lactobacillus plantarum* 299v Sanprobi IBS^®^	10 × 10^9^ CFU × 2/day, 8 weeks	HAM-D 17, SCL-90, PSS-10, APT, Stroop Test parts A and B, RFFT, TMT, CVLT, blood pro-inflammatorycytokines, kynurenines and cortisol measurements	kynurenine ↑ cognitive functions ↑	[160]
40 patients diagnosed for MDD with IBS, probiotic group (N = 20), placebo group (N = 20), randomized, double-blind, placebo controlled, multi-center, pilot clinical study	*Bacillus coagulans* MTCC 5856	2 × 10^9^ CFU/day, 90 days	HAM-D, MADRS, CES-D, IBS-QOL, CGI-I, CGI-S, RMBPC, GI-DQ, mESS, serum myeloperoxidase	depression clinical symptoms ↓ IBS symptoms ↓ sleep quality ↑ myeloperoxidase ↓	[161]
**Prebiotic studies**					
62 obese women with MDD, prebiotic group (N = 31), placebo group (N = 31), double-blind placebo-controlled randomized clinical trial	inulin	10 g/day, dissolved in a glass of water and drunk after lunch, 8 weeks	HDRS, BDI-II, biochemical parameters, anthropometric measures	no significant changes in depression symptoms weight, waist, hip circumferences, systolic blood pressure, fat mass and total cholesterol ↓ (compared to pre-intervention results, but no statistically significant effect compared to placebo)	[162]
**Probiotic and prebiotic studies**					
40 adult patients with moderate depression, symbiotic group (N = 20), placebo group (N = 20), double-blind, placebo-controlled, multi-center, randomized trial	Probiotic multispecies mixture: Familact H^®^:		HAM-D	depression clinical symptoms ↓	[163]
	*Lactobacillus casaei*	3 × 10^8^ CFU/g			
	*Lactobacillus acidofilus*	2 × 10^8^ CFU/g			
	*Lactobacillus bulgarigus*	2 × 10^9^ CFU/g			
	*Lactobacillus rhamnosus*	3 × 10^8^ CFU/g			
	*Bifidobacterium breve*	2 × 10^8^ CFU/g			
	*Bifidobacterium longum*	1 × 10^9^ CFU/g			
	*Streptococcus thermophilus*	3 × 10^8^ CFU/g			
	and fructooligosaccharide as prebiotic	100 mg			
		/capsule Patients received fluoxetine(20 mg/d) for 4 weeks before entering the study, then 2 capsules of Familact/day, for 6 weeks (plus fluoxetine)			
110 patients with MDD, probiotic group (N = 38), prebiotic group (N = 37), placebo group (N = 36), double-blind, placebo-controlled, randomized controlled trial	Probiotic: *Lactobacillus helveticus* R0052 and *Bifidobacterium longum* R0175, Prebiotic: galactooligosaccharide and 0.2% Plum flavor	Probiotic: 10 × 10^9^ CFU/ 5 g/ day Prebiotic: 5 g/ day Before a meal, by pouring the orally dispersible powder from the sachet directly into the mouth where it rapidly dissolved, 8 weeks	BDI, serum levels of kynurenine, tryptophan and BCAAs, circulating pro-inflammatory cytokine levels, urinary cortisol levels, BMI	Probiotic: depression symptoms ↓ serum kynurenine/tryptophan ratio ↓ tryptophan/ isoleucine ratio ↑ cortisol levels ↓ Prebiotic: no significant changes in depression symptoms tryptophan/BCAAs ratio ↑ cortisol levels ↓	[164,165]
**Postbiotic studies**					
60 young adult students preparing for the national examination for medical practitioners, postbiotic group (N = 29), placebo group (N = 31), double-blind, placebo-controlled, parallel-group clinical trial	Heat-inactivated *Lactobacillus gasseri* CP2305 (CP2305)	1 × 10^10^ bacterial cells pre 2 tablets/day, 24 weeks	STAI, GHQ-28, HADS, PSQI, single-channel EEG, BSS, SCFAs concentrations in feces, salivary cortisol levels, fecal microbiota analysis	anxiety ↓ sleep quality ↑ changes in microbiota composition n-valeric acid ↑	[166]
241 healthy adults, postbiotic 10LP group (N = 82), postbiotic 30LP group (N = 78), placebo group (N = 81), randomized, double-blind, placebo-controlled trial	Heat-killed *Lactobacillus paracasei* MCC1849	Two doses of *L. paracasei* MCC1849: 1 × 10^10^ heat-killed cells/day (10LP group) or 3 × 10^10^ heat-killed cells/day (30LP group), before breakfast for 12 weeks	POMS 2, TMD, incidence and severity of common cold symptoms, saliva immunoglobulin A concentration,	resistance to common cold ↑ maintaining of desirable mood state	[167]

↑: increase of the measured parameter; ↓: decrease of the measured parameter. HDRS: Hamilton depression rating scale; BDI-II: Beck depression inventory, CAN-BIND: Canadian Biomarker Integration Network in Depression, MADRS: Montgomery–Åsberg Depression Rating Scale; QIDS-SR16: Quick Inventory of Depressive Symptomatology; SHAPS: Snaith–Hamilton Pleasure Scale, GAD-7: Generalized Anxiety Disorder 7-item scale; STAI: State-Trait Anxiety Inventory, PSQI: Pittsburgh Sleep Quality Index; EPDS: Edinburgh Postnatal Depression Scale; LEIDS-R: Leiden Index of Depression Sensitivity-Revised; PRAQ-R: Pregnancy-Related Anxiety Questionnaire-Revised; PES: Pregnancy Experience Scale; MAAS: Maternal Antenatal Attachment Scale); MPAS: Maternal Postnatal Attachment Scale; HAM-D 17: Hamilton Depression Rating; SCL-90: Symptom Checklist; PSS-10: Perceived Stress Scale; APT: Attention and Perceptivity Test; RFFT: Ruff Figural Fluency Test; TMT: Trail Making Test; CVLT: California Verbal Learning Test; BCAAs: Branch chain amino acids; CES-D: Centre for Epidemiological Studies–Depression Scale; IBS-QOL: Irritable Bowel Syndrome Quality of Life; CGI-I: Clinical Global Impression-Improvement Rating Scale; CGI-S: Clinical Global Impression Severity Rating Scale; RMBPC: Revised Memory and Behavior Problem Checklist; GI-DQ: Gastrointestinal Discomfort Questionnaire; mESS: Modified Epworth Sleepiness Scale; STAI: Spielberger State-Trait Anxiety Inventory; GHQ-28: 28-item General Health Questionnaire; HADS: Hospital Anxiety and Depression Scale; BSS: Bristol Stool Scale; POMS 2: Profile of Mood States; TMD: total mood disturbance scale; MDD: major depressive disorder.

## Data Availability

Not applicable.
